# Age-specific climate sensitivity of respiratory hospitalizations in a tropical coastal city: 20-year Random Forest forecasts

**DOI:** 10.1007/s00484-026-03284-4

**Published:** 2026-08-03

**Authors:** Marcos Paulo Santos Pereira, Rafaela Lisboa Costa, Fabricio Daniel dos Santos Silva, Glauber Lopes Mariano, Heliofabio Barros Gomes, Helber Barros Gomes, Djane Fonseca da Silva, João Otávio Alves Accioly, Jean Souza dos Reis, Eva Maria Mollinedo Veneros, Bruno Coelho Bulcão, Marcelo Félix Alonso, Flavio Manoel Rodrigues da Silva Júnior, Richard James Ladle, Fabiana Rita do Couto Santos Pereira

**Affiliations:** 1 Institute of Atmospheric Sciences, Federal University of Alagoas, Maceió, Brazil; 2 Graduate Program in Environmental Engineering, Federal University of Technology, Londrina, Brazil; 3 Faculty of Meteorology, Federal University of Pelotas, Pelotas, Brazil; 4 Institute of Biological and Health Sciences, Federal University of Alagoas, Maceió, Brazil; 5 Climate Change and Energy Center, Ayni Institute: Environmental Conservation and Social Development, Maceió, Brazil

**Keywords:** Respiratory diseases, Climate–health interactions, Tropical climate, Machine learning, Lag effects

## Abstract

Respiratory diseases remain a major public health challenge in tropical coastal cities, where persistent heat-humidity interactions and climate variability shape population vulnerability. This study quantified associations between atmospheric conditions and respiratory hospitalizations in Maceió, Brazil, using a 20-years time series (2000–2019). Weekly hospitalization rates stratified by age (children 0–4 years, adults 5–59 years, elderly ≥ 60 years), were modeled against meteorological variables—including temperature, relative humidity, precipitation, atmospheric pressure, and solar radiation—considering lag structure of 0, 1, and 2 weeks. Random Forest regression models were applied to capture nonlinear relationships and forecast hospitalization rates. Minimum temperature was the dominant predictor, exhibiting strong inverse associations across all age groups (ρ = − 0.65, p < 0.001), with effects persisting up to two weeks. Age-specific patterns were observed: children showed immediate sensitivity to thermal and precipitation variables, whereas elderly populations exhibited delayed responses to barometric pressure and evaporation. Model performance was high for children and adults (R^2^ = 0.83–0.90) and moderate for the elderly, with Symmetric Mean Absolute Percentage Error ranging from 13 to 25% across groups. Long-term trends revealed declining hospitalization rates among children and adults, contrasted by stabilization and subsequent increases in the elderly after 2010, consistent with demographic aging and increased climate sensitivity. These findings demonstrate the value of machine learning approaches for modeling complex climate-health relationships and provide a transferable framework for climate-informed respiratory risk assessment and early warning systems in tropical coastal environments.

## Introduction

Respiratory diseases remain a major global public health concern, contributing substantially to morbidity, mortality, and disability-adjusted life years worldwide. The Global Burden of Disease Study 2019 estimated more than 100 million annual incident cases of lower respiratory infections, resulting in approximately 2.6 million deaths (GBD 2019 Diseases and Injuries Collaborators 2020). Although mortality rates have declined in recent decades, the burden remains disproportionately high in low- and middle-income countries, where structural inequalities, environmental exposures, and limited healthcare access exacerbate vulnerability (World Health Organization 2023; Landrigan et al. 2018). Poor housing conditions, high population density, and exposure to climatic extremes further amplify respiratory morbidity and reinforce health inequities (Ebi et al. 2021).

Chronic respiratory diseases affect an estimated 545 million individuals globally (Soriano et al. 2020), and many are highly sensitive to climatic variability. Hospital admissions and symptom exacerbations have been linked to temperature extremes, elevated humidity, heavy precipitation, and abrupt weather transitions (Li et al. 2022; Silva et al. 2014; Xu et al. 2020). Both heat and cold exposure are associated with increased respiratory hospitalizations and mortality, particularly during extreme events such as heatwaves (Achebak et al. 2023; Gasparrini et al. 2015). High humidity can impair thermoregulation and mucociliary clearance while enhancing pathogen survival, and intense rainfall promotes mold growth and aeroallergen dispersion, further aggravating respiratory conditions (Duan et al. 2021; Wu et al. 2016; World Health Organization 2020).

These climate–health interactions are especially pronounced in tropical coastal cities, where persistent heat-humidity coupling and limited nocturnal cooling create conditions of chronic thermal stress. In environments such as Maceió, northeastern Brazil, sustained relative humidity exceeding 80% and marine aerosol exposure may intensify airway inflammation and respiratory vulnerability (Field et al. 2021; Edwards et al. 2021). Rising wet-bulb temperatures, often exceeding 29 °C in urban areas, represent an additional physiological stressor, particularly for children and older adults (Raymond et al. 2020; Mora et al. 2017). Despite these risks, relatively few climate–health modeling studies focus on tropical coastal settings, leaving important gaps in understanding continuous exposure to compound heat and humidity (de Schrijver et al. 2021).

Addressing these gaps requires locally contextualized analyses that integrate long-term meteorological variability with demographic and health data. In this study, we employed a retrospective ecological time-series design to examine associations between atmospheric conditions and respiratory disease hospitalizations in Maceió over a 20-year period (2000–2019). Using a Random Forest framework, we developed predictive models to forecast weekly hospitalization rates while evaluating age-specific vulnerability patterns. This approach provides a transferable basis for climate-informed respiratory risk assessment and early warning systems in tropical coastal environments.

Despite growing evidence on climate–health interactions, important gaps remain in understanding age-specific respiratory risks in tropical coastal environments, where persistent heat–humidity conditions and distinct seasonal dynamics differ substantially from temperate regions. Most existing studies focus on short-term associations, single age groups, or rely on linear modeling approaches that may not adequately capture complex lagged and nonlinear relationships between atmospheric variables and health outcomes.

In this context, the present study aims to quantify the age-specific climate sensitivity of respiratory hospitalizations in a tropical coastal city over a 20-year period. Using a Random Forest framework, we model nonlinear and lagged associations between multiple meteorological drivers and respiratory outcomes. This approach enables the identification of key predictors, characterization of age-specific lag structures, and advancement of climate-informed respiratory risk assessment. By integrating long-term epidemiological and atmospheric data, this study provides novel insights into differential vulnerability across age groups and contributes to the development of predictive tools for early warning systems in underrepresented low-latitude settings.

## Materials and methods

### Study area and design

This retrospective ecological time-series study was conducted in Maceió (09°39′S, 35°44′W), capital of Alagoas State, northeastern Brazil. The city is characterized by a tropical coastal climate with persistently high temperatures (annual mean ~ 25.5 °C), elevated relative humidity (~ 80%), and a pronounced rainy season from March to May. The study period spanned from 1 January 2000 to 31 December 2019, enabling assessment of short-term meteorological effects and long-term climate–health interactions under evolving demographic conditions.

### Health, population, and meteorological data

Hospitalization data were obtained from the Hospital Information System of the Brazilian Unified Health System (SIH/SUS), accessed via DATASUS. Records were restricted to respiratory diseases (ICD-10 codes J00–J99) and aggregated by date of admission, municipality of residence, and age group. Three age categories were defined: children (0–4 years), adults (5–59 years), and elderly (≥ 60 years), allowing evaluation of age-specific vulnerability patterns, consistent with evidence of differential climatic sensitivity across life stages (Gasparrini et al. 2015).

Annual population estimates were retrieved from the Brazilian Institute of Geography and Statistics (IBGE), and age-specific weekly denominators were derived using linear interpolation between census years. Weekly hospitalization rates per 100,000 inhabitants were calculated to ensure temporal and demographic comparability.

Daily meteorological data for the same period were obtained from the National Institute of Meteorology (INMET), including minimum, mean, and maximum air temperature (°C), relative humidity (%), atmospheric pressure (hPa), wind speed (m s⁻^1^), solar radiation (h), evaporation (mm), and precipitation (mm). The selection for meteorological variables was based on both biometeorological relevance and data completeness thoughout the study period. Variables with the most consistent temporal coverage between 2000 and 2019 were prioritized to ensure robustness of long-term analyses. The selected parameters represent key atmospheric dimensions associated with respiratory health in tropical coastal environments, including thermal conditions (temperature), moisture dynamics (humidity, precipitation, evaporation), barometric variability (atmospheric pressure), and atmospheric exchange processes (wind speed and solar radiation), all of which have been associated with respiratory disease dynamics in previous climate–health studies (Gasparrini et al. 2015; Tamerius et al. 2011; Mendell et al. 2011). Prior to analysis, meteorological datasets were inspected for completeness, plausibility, and temporal consistency. Quality control procedures included removal of implausible values and temporal alignment with hospitalization records. Missing observations were treated using linear interpolation for gaps of ≤ 3 consecutive days and multiple imputation for longer gaps, following established recommendations for environmental time-series analysis (Little and Rubin 2019). Daily observations were subsequently aggregated into weekly means (temperature, humidity, pressure, wind speed, radiation) or weekly totals (precipitation, evaporation) to ensure temporal compatibility with weekly hospitation outcomes.

Population data were obtained from the Brazilian Institute of Geography and Statistics (IBGE) and included annual age-stratified population estimates. Weekly population denominators were derived using linear interpolation between census years (2000 and 2010) and intercensal estimates to ensure temporal consistency with hospitalization records. Meteorological data were obtained from the National Institute of Meteorology (INMET) and aggregated to weekly averages or cumulative values, depending on the variable. All datasets were temporally aligned at the weekly level to ensure consistency between health, demographic, and atmospheric variables.

Meteorological variables were selected to represent key dimensions of atmospheric conditions relevant to respiratory health, including thermal (temperature), moisture (relative humidity, precipitation, and evaporation), barometric (atmospheric pressure), and radiative (solar radiation) processes. These variables influence respiratory outcomes through multiple pathways, including airway reactivity, pathogen transmission dynamics, indoor environmental conditions, and allergen exposure. The inclusion of multiple complementary variables enables a more comprehensive assessment of climate–health interactions compared to single-variable approaches. Lagged exposures (0–2 weeks) were incorporated to account for delayed physiological responses, incubation periods, and healthcare-seeking behavior.

To account for delayed physiological and epidemiological responses, meteorological variables were evaluated at three lag structures: concurrent week (lag 0), 1-week delay (lag 1), and 2-week delay (lag 2). This approach captures delayed climatic effects and typical incubation periods associated with respiratory infections (Xu et al. 2013; Hajat et al. 2024).

### Statistical analysis and predictive modeling

Spearman rank-order correlation coefficients (ρ) were computed between weekly meteorological variables and age-stratified hospitalization rates at each lag (0, 1, and 2 weeks). Lag structures of 0, 1, and 2 weeks were selected based on plausible biological and epidemiological mechanisms linking atmospheric conditions to respiratory morbidity. Lag 0 was considered to capture immediate effects of thermal stress and airway reactivity, whereas lag 1 reflects typical incubation periods for common respiratory viral infections, including influenza and respiratory syncytial virus. Lag 2 was included to account for delayed healthcare-seeking behavior, cumulative exposure effects, and prolonged physiological responses reported in climate–respiratory studies. To reduce the likelihood of type I error associated with multple comparisons, Bonferroni correction was applied (α = 0.005). Correlation analyses were used as exploratory measures of association and should not be interpreted as evidence of causality.

Predictive modeling was conducted using Random Forest regression to estimate weekly respiratory hospitalization rates for each age group. Random Forest was selected due to its capacity to model nonlinear exposure–response relationships, accommodate multicollinearity among meteorological predictors, and capture complex interactions between atmospheric conditions and temporal dynamics without requiring parametric assumptions. Recent environmental forecasting studies have also demonstrated strong predictive performance of Random Forest models in climate-related applications in northeastern Brazil (Reis et al. 2025), supporting their use in ecological forecasting contexts.

Data partitioning followed a time-aware strategy preserve temporal structure and avoid information leakage, with observation from 2000–2018 used for model development and internal validation, and 2019 reserved as an independent hold-out year for external evaluation. Hyperparameters were optimized through grid search with time-aware fivefold cross-validation applied exclusively to the training period. The search procedure evaluated combinations of maximum tree depth (max_depth), minimum samples required for node splitting (min_samples_split), and minimum samples per leaf (min_samples_leaf).

Final Random Forest models were configured with 500 trees (n_estimators = 500), out-of-bag estimation enabled for internal consistency assessment (oob_score = True), unrestricted maximum tree depth (max_depth = None), minimum samples required for node splitting equal to 2 (min_samples_split = 2), and minimum samples per leaf equal to 1 (min_samples_leaf = 1). Other parameters followed the default Scikit-learn implementation. Out-of-bag error was monitored as an additional internal estimate of predictive performance and model stability. An exhaustive hyperparameter optimization strategy was not pursued because the primary objective of the study was ecological forecasting and interpretation of climate–health relationships rather than algorithmic optimization.

Model performance was evaluated using mean absolute error (MAE), root mean square error (RMSE), coefficient of determination (R^2^), and symmetric mean absolute percentage error (SMAPE), selected for its robustness and interpretability in forecasting applications (Hyndman and Koehler 2006). All analyses were performed in Python 3.8 using Pandas, NumPy, SciPy, and Scikit-learn.

## Results

### Meteorological conditions and temporal hospitalization patterns

Meteorological conditions in Maceió during 2000–2019 were characterized by persistently high temperatures (mean maximum: 29.9 °C) and elevated relative humidity (mean: 80.1%; 75th percentile: 84%), with minimal diurnal thermal variation (minimum temperature median: 21.9 °C) (Table 1). Precipitation exhibited marked variability (maximum: 187.8 mm), whereas atmospheric pressure remained relatively stable (mean: 1004.9 hPa).

**Table 1:** Descriptive statistics of meteorological variables in Maceió, Brazil (2000–2019). Data include mean, minimum (Min), maximum (Max), median, and 25th/75th percentiles (P25/P75) for temperature, humidity, wind speed, solar radiation, evaporation, precipitation, and atmospheric pressure

Variable	Mean	Min	Max	Median	P25	P75
Min. Temperature (°C)	21.58	15	26	21.9	20.4	22.9
Max. Temperature (°C)	29.91	23	36.8	30	28.5	31.4
Mean Temperature (°C)	25.54	20.1	30.1	25.6	24.5	26.6
Mean Humidity (%)	80.08	57.5	99	79.8	75.8	84
Min Humidity (%)	69.79	45	97	69	64	75
Wind Speed (m s^−1^)	2.71	0	7.8	2.6	2.1	3.3
Radiation (h)	6.74	0	12	7.4	4.6	9.3
Evaporation (mm)	2.84	0	13	2.8	2.3	3.2
Precipitation (mm)	5.23	0	187.8	0.2	0	5.3
Pressure (hPa)	1004.94	998	1012.6	1004.7	1003.3	1006.6

**Fig. 1:**
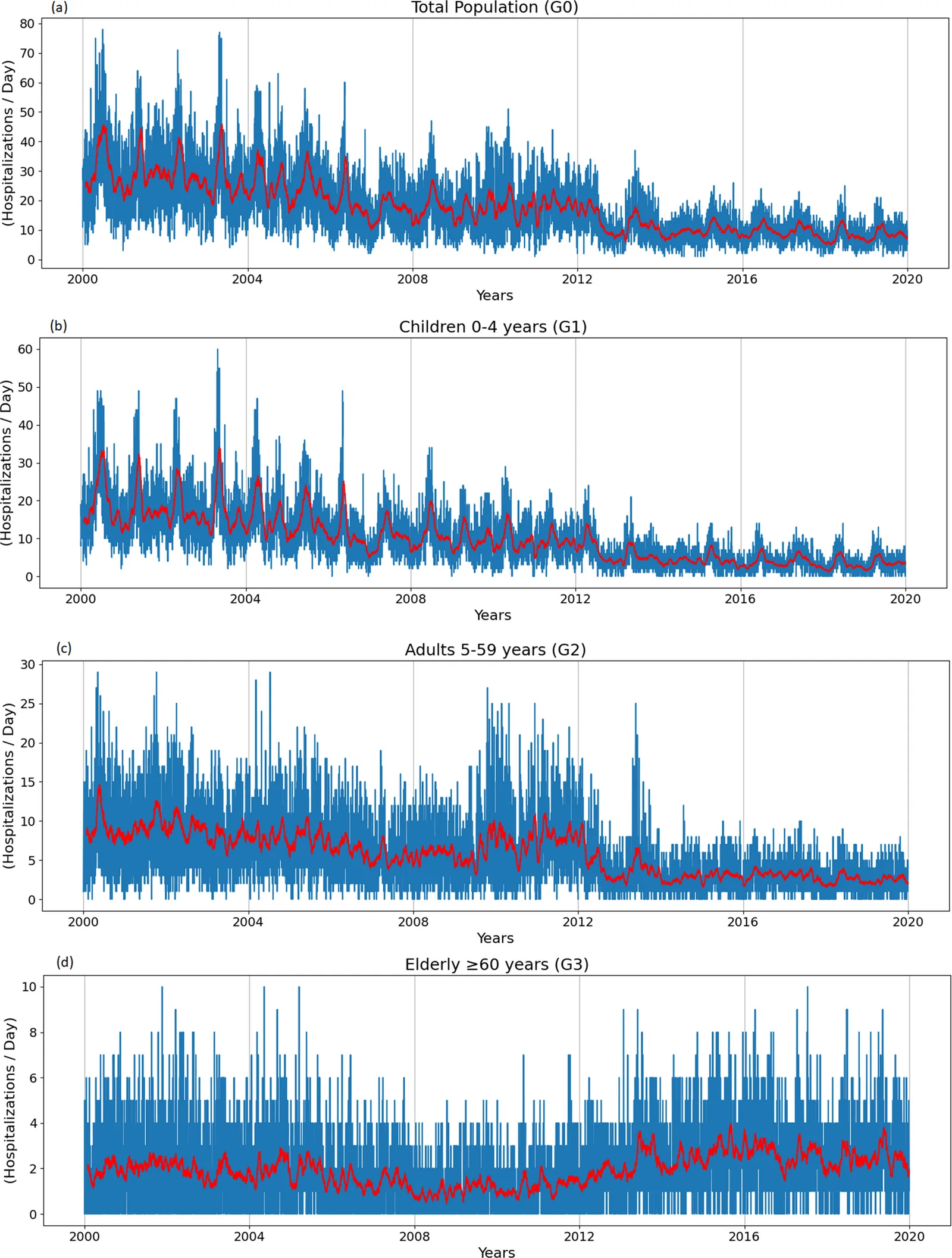
Daily respiratory disease hospitalizations in Maceió, Brazil (2000–2019), showing long-term temporal trends by age group: (**a**) total population (G0); (**b**) children aged 0–4 years (G1); (**c**) adults aged 5–59 years (G2); and (**d**) elderly aged ≥ 60 years (G3). Black lines represent observed daily hospitalizations, and the red line denotes the 30-day moving average. The figure highlights long-term trends, including marked declines in 2006 and 2012 and divergent trajectories among age groups over the study period. Data source: DATASUS

The 20-year time series revealed a significant declining trend in respiratory hospitalizations in the total population (− 1.24 hospitalizations/day/year; 95% CI: − 1.42 to − 1.03), corresponding to a cumulative reduction of approximately 24.8 hospitalizations/day (Fig. 1a). Structural declines in 2006 and 2012 coincided with expansion of the Family Health Strategy and pneumococcal vaccination programs (Domingues et al. 2020; Macinko and Harris 2015).

Age-stratified analyses showed heterogeneous trajectories. Children exhibited the strongest decline (− 0.92/day/year; 95% CI: − 1.03 to − 0.79) and the highest seasonal amplitude (Fig. 1b), adults demonstrated a moderate reduction (− 0.38/day/year; 95% CI: − 0.44 to − 0.31) (Fig. 1c), and the elderly showed no significant overall trend (+ 0.04/day/year; 95% CI: − 0.02 to + 0.09), with a downward phase (2000–2009) followed by an upward trajectory (2010–2019), consistent with demographic aging and increased climate sensitivity (Miranda et al. 2016; Silva et al. 2020) (Fig. 1d).

**Fig. 2:**
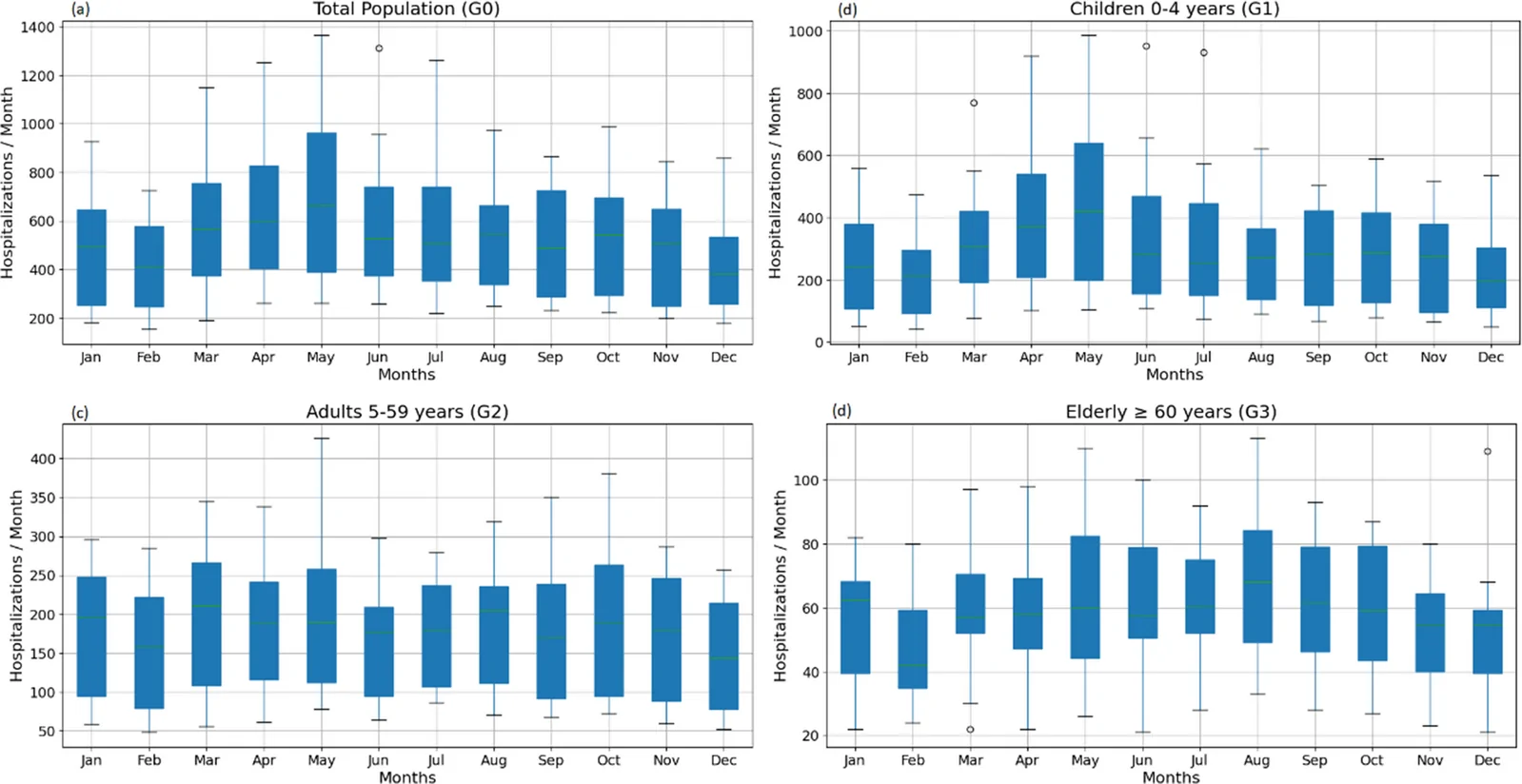
Seasonal distribution of daily respiratory disease hospitalizations in Maceió, Brazil (2000–2019), by age group: (**a**) total population (G0); (**b**) children aged 0–4 years (G1); (**c**) adults aged 5–59 years (G2); and (**d**) elderly aged ≥ 60 years (G3). Curves illustrate intra-annual variability, highlighting peak hospitalizations during March–May (rainy season) for most groups and a delayed seasonal peak (August–September) among the elderly. The red line denotes the 30-day moving average. Data source: DATASUS

Pronounced seasonality was observed across all groups (Fig. 2), with a unimodal peak during March–May (rainy season) in the total population and children. Seasonal variability increased by 23% in the total population and 18% in children, based on interquartile range (IQR) estimates, a robust dispersion metric (Wilcox 2017). Adults displayed more distributed peaks (March, August, January), whereas the elderly showed delayed maxima in August–September.

### Meteorological correlates and lag structure

**Fig. 3:**
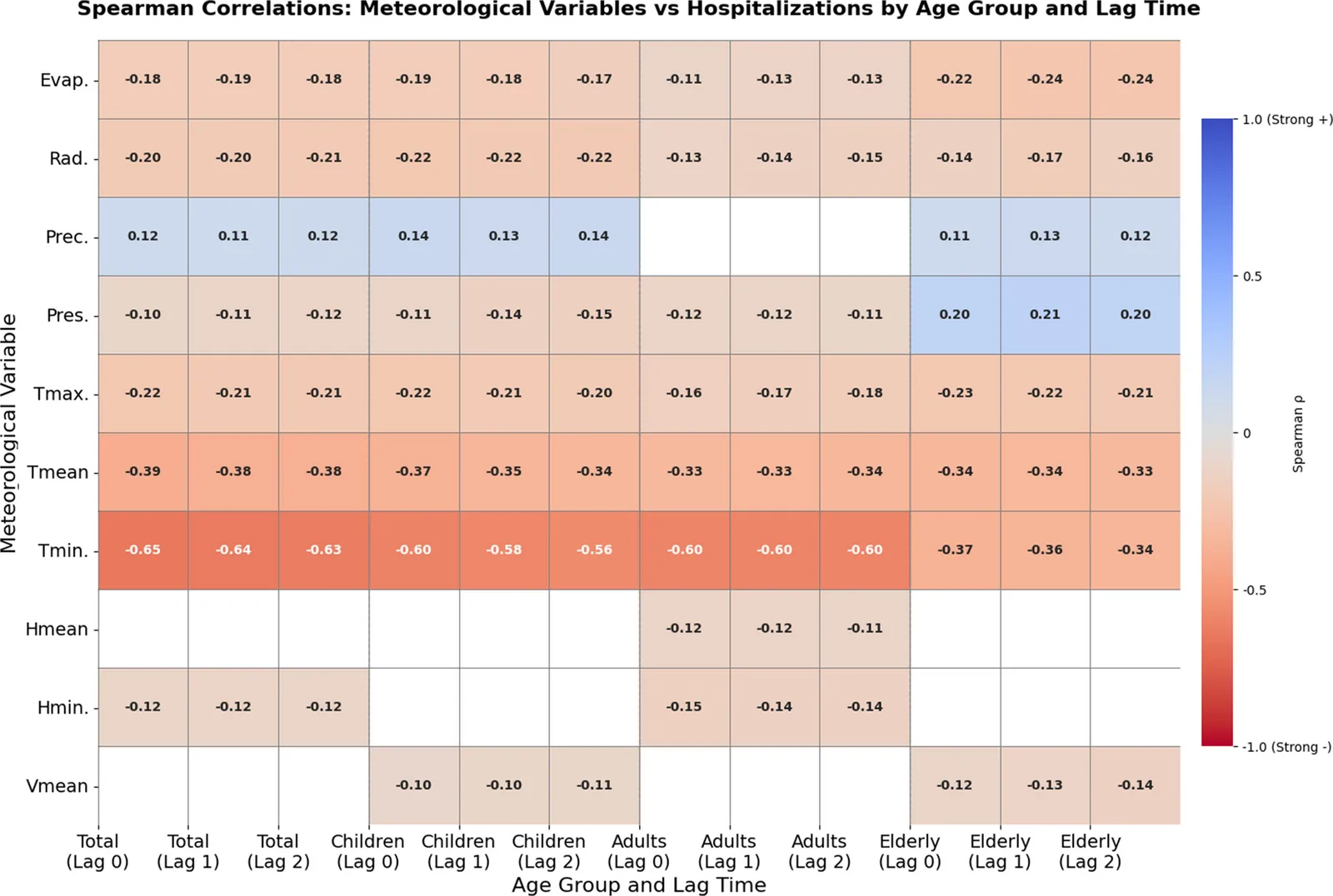
Spearman correlations (ρ) between meteorological variables and age-stratified hospitalization rates at lag 0 (same week), lag 1 (1-week delay) and lag 2 (2-week delay). Color intensity reflects ρ values (blue: positive, red: negative). White background indicates non-significant associations (*p* ≥ 0.005 Bonferroni-adjusted)

Spearman analyses demonstrated consistent age-specific and lag-dependent associations between meteorological variables and hospitalization rates (Fig. 3).

At lag 0, minimum temperature exhibited the strongest inverse correlation in the total population (ρ = − 0.654, p < 0.001), followed by mean temperature and solar radiation, while precipitation showed weak positive associations. Children mirrored this temperature sensitivity (ρ = − 0.602), with stronger precipitation effects than adults. Adults showed greater sensitivity to minimum humidity, whereas the elderly exhibited distinct associations with mean humidity and atmospheric pressure.

At lag 1, correlation magnitudes remained comparable, with peak atmospheric pressure effects in the elderly (ρ = + 0.215, p < 0.001) and pronounced inverse associations with evaporation. At lag 2, correlations weakened but remained significant; minimum temperature continued to show strong inverse associations across groups, and pressure and evaporation effects persisted in the elderly, suggesting cumulative environmental stress.

Overall, the results indicate heterogeneous temporal responses to meteorological exposures, with minimum temperature emerging as the dominant predictor across age groups and pressure-related variables showing particular relevance in older adults.

### Random Forest performance and validation

**Fig. 4:**
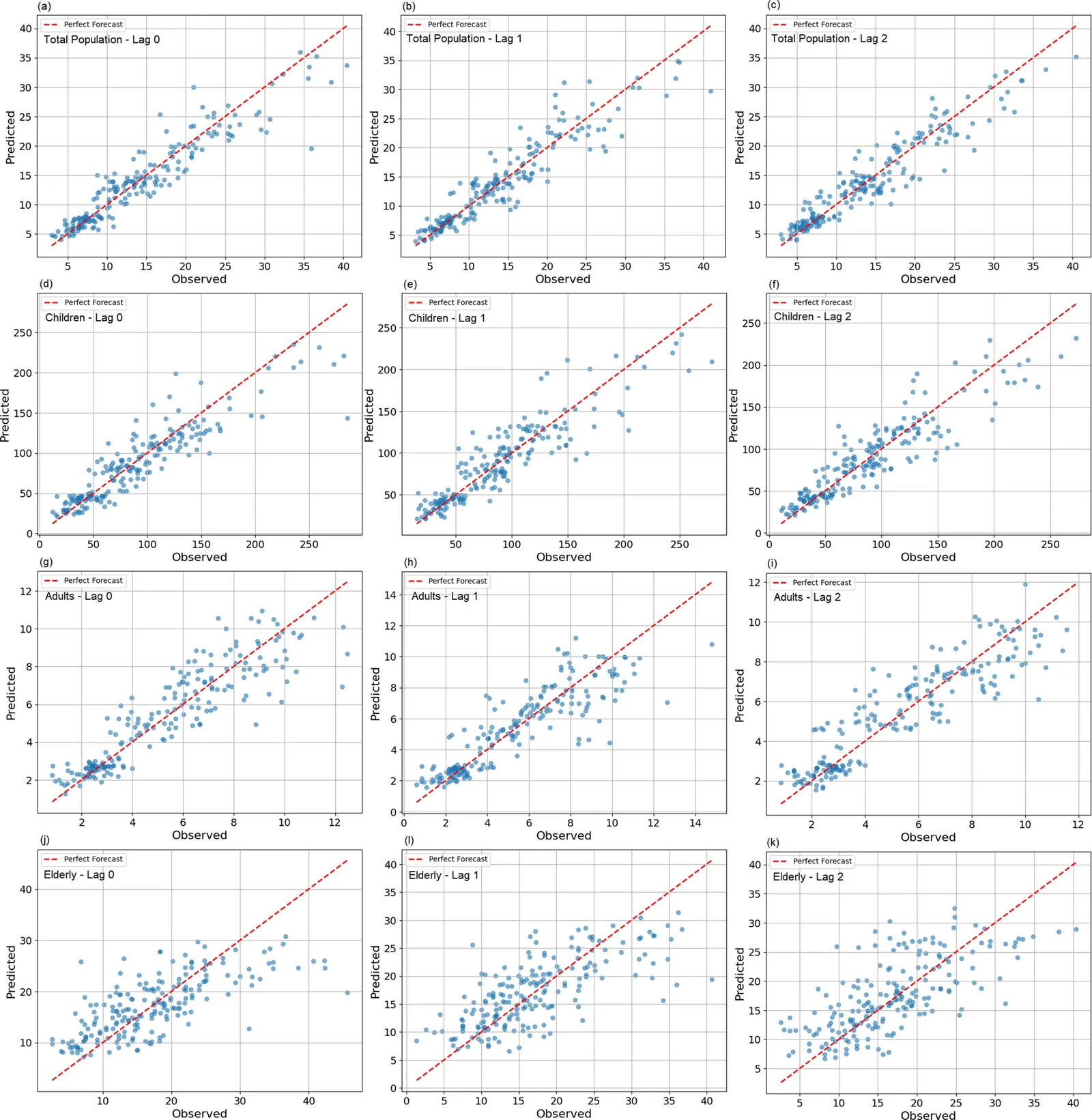
Predicted versus observed weekly respiratory hospitalization rates (per 100,000 inhabitants) in Maceió, Brazil, from 2000 to 2018, using the Random Forest model. Panels are organized by age group (rows) and exposure–response lag period (columns). Rows represent: (G0) Total population (**4a**–**c**), (G1) Children aged 0–4 years (**4d**–**f**), (G2) Adults aged 5–59 years (4** g**–**i**), and (G3) Elderly aged ≥ 60 years (**4j**–**l**). Columns correspond to lag intervals between meteorological exposure and hospitalizations: lag 0 (same week; **4a**,**d**,**g**,**j**), lag 1 (1-week delay; **4b**,**e**,**h**,**k**), and lag 2 (2-week delay; **4c**,**f**,**i**,**l**)

Random Forest models demonstrated strong predictive capacity (Table 2; Fig. 4). For the total population, the highest performance occurred at lag 2 (R^2^ = 0.90; RMSE = 2.49), indicating delayed meteorological effects. Children showed consistent performance across lags (R^2^ = 0.82–0.83), while adults achieved optimal predictions at lag 2 (R^2^ = 0.83; MAE = 0.95). Performance in the elderly was moderate (R^2^ = 0.45–0.51), likely reflecting additional clinical and behavioral determinants not captured by meteorological predictors.

**Table 2:** Random Forest model performance metrics (training/testing: 2000–2018)

Group	Lag	MAE	RMSE	R^2^
Total Population	0	1.89	2.74	0.88
Total Population	1	1.88	2.71	0.87
Total Population	2	1.87	2.49	0.90
Children	0	15.98	22.98	0.82
Children	1	15.61	22.08	0.82
Children	2	16.34	22.05	0.83
Adults	0	0.89	1.22	0.81
Adults	1	1.01	1.41	0.77
Adults	2	0.95	1.22	0.83
Elderly	0	4.52	5.91	0.51
Elderly	1	4.16	5.34	0.45
Elderly	2	4.15	5.41	0.51

**Fig. 5:**
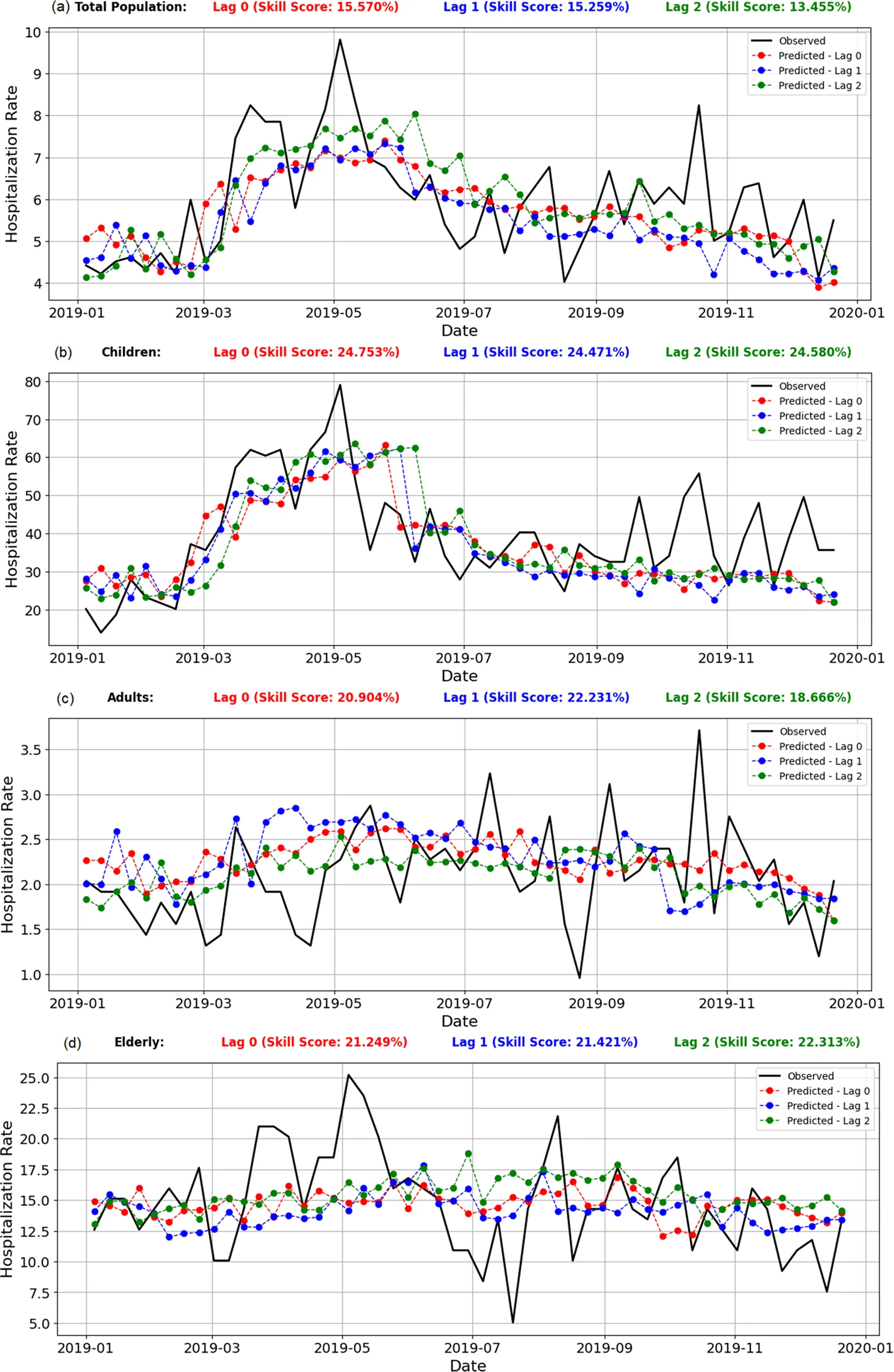
Comparison between observed and predicted weekly respiratory hospitalization rates in Maceió, Brazil, for the independent validation year 2019. Panels correspond to age groups: (**5a**) total population (G0), (**5b**) children aged 0–4 years (G1), (**5c**) adults aged 5–59 years (G2), and (**5d**) elderly aged ≥ 60 years (G3). The black line represents observed hospitalization rates, while colored lines indicate Random Forest model predictions using different exposure–response lag structures: red for lag 0 (same-week meteorological exposure), blue for lag 1 (1-week delay), and green for lag 2 (2-week delay). The figure illustrates the model’s ability to capture temporal variability and age-specific dynamics in respiratory disease hospitalizations, highlighting differences in predictive accuracy across lag periods and population groups

Independent validation using 2019 data confirmed robust predictive accuracy (Fig. 5). Forecast skill, assessed using SMAPE (Hyndman and Koehler 2006), indicated excellent performance (< 20%) for the total population across all lags, with progressive improvement at lag 2 (13.46%). Children and adults showed good-to-excellent performance, particularly at lag 2, supporting delayed-response hypotheses. The elderly maintained good predictive skill (~ 21–22%), despite greater complexity.

All SMAPE values were within excellent-to-good ranges (< 25%), confirming the stability and generalizability of the age-stratified, lag-structured forecasting framework for respiratory disease surveillance in tropical coastal environments.

## Discussion

Respiratory disease hospitalizations in Maceió were consistently modulated by local atmospheric conditions, with clear age-specific and lag-dependent responses. The long-term decline observed among children and adults aligns with national public health improvements in Brazil, including the expansion of primary care and vaccination programs (Victora et al. 2011; Barreto et al. 2011; Macinko and Harris 2015; Domingues et al. 2020) In contrast, the stabilization and subsequent increase in hospitalizations among the elderly after approximately 2010 reflects demographic aging and increased vulnerability to environmental stressors (Miranda et al. 2016; Silva et al. 2020; Guimarães et al. 2021). This inflection is consistent with the broader phenomenon of stalling mortality trends observed in several countries after 2010–2012, suggesting a convergence of demographic and epidemiological transitions that may amplify climate sensitivity in older populations (Ho and Hendi 2018; Raleigh 2019).

Seasonal patterns in Maceió differ from temperate regions, with a pronounced peak during the rainy season rather than winter. This finding reinforces the need to reinterpret classical frameworks such as excess winter mortality (EWM) in tropical contexts, where excess morbidity may instead be associated with periods of increased precipitation and humidity. Recent studies have also highlighted long-term variability and cyclic patterns in seasonal mortality across different regions (Walkowiak and Walkowiak 2024; Walkowiak et al. 2025). Although the present study focuses on hospitalizations rather than mortality, the concept of “excess rainy-season morbidity” provides a useful perspective for understanding seasonal amplification of respiratory risk.Previous studies have linked increased rainfall and humidity to enhance viral transmission, indoor crowding, and environmental exposure to dampness and allergens (Tamerius et al. 2011; Chowdhury et al. 2018). Emerging evidence also suggests that precipitation variability can influence infectious disease dynamics in tropical regions, reinforcing the climate sensitivity of respiratory outcomes.

### Meteorological drivers and physiological pathways

Minimum temperature emerged as the most consistent predictor across all age groups, with inverse associations persisting up to two-week lags. This supports the hypothesis that, in tropical environments, relative thermal variability rather than extreme cold plays a key role in triggering respiratory morbidity. The use of a Random Forest framework allowed the identification of these nonlinear and lagged relationships, which may not be fully captured by traditional linear models. The importance of minimum temperature across age groups highlights its role in modulating airway reactivity, immune responses, and viral stability under humid tropical conditions (Gasparrini et al. 2015; Zhao et al. 2019).

Moisture-related variables, including precipitation, humidity, and evaporation, showed consistent positive associations, particularly among children and the elderly. These findings are consistent with mechanisms involving indoor dampness, fungal proliferation, and allergen exposure (Mendell et al. 2011; Harley et al. 2009). In coastal urban settings such as Maceió, where housing inadequacies—including poor ventilation and moisture intrusion—remain prevalent in lower-income communities, these environmental exposures may amplify the health impacts of climatic variability. Additionally, the observed sensitivity to atmospheric pressure and evaporation among the elderly suggests increased vulnerability to compound environmental stressors, likely reflecting reduced physiological resilience.

### Predictive modeling and climate implications

The predictive performance of the Random Forest models (R^2^ > 0.80 in most groups) demonstrates the value of machine learning approaches for modeling complex climate–health interactions. Improved performance at longer lags supports the role of delayed physiological responses, incubation periods, and healthcare-seeking behavior (Hajat et al. 2024; Xu et al. 2013). Nevertheless, the more moderate performance observed in the elderly indicates that meteorological predictors alone may not fully explain variability in this group, reinforcing the importance of clinical and social determinants. In addition, while visual inspection of temporal trends suggests potential nonlinear dynamics, linear approximations were retained to ensure interpretability and comparability across groups, representing a balance between model complexity and analytical clarity.An additional consideration relates to the “time-to-death” effect, whereby a substantial proportion of healthcare utilization occurs in the final year of life. Although the ecological design of this study does not allow linkage between hospitalization and mortality records, this phenomenon may partially contribute to the elevated hospitalization burden observed among the elderly and should be explored in future studies using individual-level data.

More broadly, the climatic regime of Maceió—characterized by sustained heat, high humidity, and recurrent rainfall—represents a context of continuous environmental exposure rather than episodic extremes. This study contributes to addressing a critical gap in climate–health research by providing long-term, age-stratified evidence from a tropical coastal setting, where nonlinear and interacting atmospheric drivers shape respiratory risk. These findings support the development of climate-informed early warning systems and highlight the need for locally calibrated models that account for compound climatic stressors.

### Public health implications and study limitations

The identified age-specific sensitivities and lag structures support integration of meteorological surveillance into early warning systems for respiratory diseases in tropical coastal cities. Forecast-based alerts 1–2 weeks in advance, hospital surge planning, and anticipatory vaccination or risk communication strategies could enhance climate-resilient health planning.

The identified lag structures also provide a potential operational framework for climate-informed early warning systems. Forecasts indicating abrupt reductions in minimum temperature or periods of intense precipitation could support anticipatory public health actions tailored to vulnerable age groups. For example, pediatric populations may benefit from earlier preventive alerts during rainy-season periods, whereas elderly populations may require longer lead times due to delayed hospitalization responses and increased vulnerability to cumulative climatic stressors.

Despite these contributions, this study has limitations inherent to ecological designs, including the inability to infer individual-level causality. Hospitalization data capture severe cases and may underestimate the total burden of respiratory disease in the community. Additionally, the absence of air pollution measurements may limit interpretation of the isolated contribution of meteorological variables, given the well-established interactions between air quality and respiratory outcomes (Silva et al. 2014).

Although Maceió provides an important example of a tropical coastal environment characterized by persistent heat–humidity exposure and marked rainy-season dynamics, caution is warranted when extrapolating these findings to other low-latitude regions. Differences in urban infrastructure, socioeconomic conditions, healthcare systems, demographic structure, and regional climatic regimes may modify climate–health relationships across tropical settings. Replication studies in cities with distinct environmental and social profiles are therefore necessary to assess the broader generalizability of the observed associations and predictive patterns.

Socioeconomic heterogeneity and disparities in healthcare access across urban sectors of Maceió were also not explicitly incorporated into the analyses. Variations in housing quality, population density, access to primary healthcare, and healthcare-seeking behavior may influence both exposure and hospitalization risk, particularly among socially vulnerable populations. Future research should integrate environmental, clinical, socioeconomic, and healthcare accessibility indicators within multilevel or spatially explicit frameworks to improve understanding of climate-sensitive respiratory vulnerability in tropical coastal cities, particularly among aging populations.

## Conclusions

This study demonstrates that respiratory hospitalizations in a tropical coastal city are strongly influenced by atmospheric conditions, with clear age-specific and lag-dependent responses. Minimum temperature and moisture-related variables emerged as key drives, particularly affecting children and elderly population under persistent heat-humidity conditions.By applying a machine learning framework to long-term data, this study advances current understanding beyond traditional seasonal analyses, highlighting the importance of nonlinear and delayed climate–health relationships in underrepresented tropical environments. These findings contribute to filling a critical gap in the biometeorology literature.

From a public health perspective, the results support the integration of meteorological information into early warning systems and age-targeted intervention strategies, particularly in rapidly aging populations exposed to compound climatic stressors.

## Data Availability

The datasets analyzed during the current study are publicly available from DATASUS (Brazilian Ministry of Health) and INMET meteorological databases.
